# Epidemiology, Risk Factors and Measures for Preventing Drowning in Africa: A Systematic Review

**DOI:** 10.3390/medicina55100637

**Published:** 2019-09-25

**Authors:** Lauren Miller, Faith O. Alele, Theophilus I. Emeto, Richard C. Franklin

**Affiliations:** 1 Public Health and Tropical Medicine, College of Public Health, Medical and Veterinary Sciences, James Cook University, Townsville, QLD 4811, Australia; lauren.miller4@my.jcu.edu.au (L.M.); theophilus.emeto@jcu.edu.au (T.I.E.); richard.franklin@jcu.edu.au (R.C.F.); 2 College of Healthcare Sciences, James Cook University, Townsville, QLD 4811, Australia

**Keywords:** drowning, immersion injuries, Africa

## Abstract

*Background and Objectives*: Drowning is a leading cause of unintentional injury related mortality worldwide, and accounts for roughly 320,000 deaths yearly. Over 90% of these deaths occur in low- and middle-income countries with inadequate prevention measures. The highest rates of drowning are observed in Africa. The aim of this review is to describe the epidemiology of drowning and identify the risk factors and strategies for prevention of drowning in Africa. *Materials and Methods*: A review of multiple databases (MEDLINE, CINAHL, PsycINFO, Scopus and Emcare) was conducted from inception of the databases to the 1st of April 2019 to identify studies investigating drowning in Africa. The preferred reporting items for systematic review and meta-analysis (PRISMA) was utilised. *Results*: Forty-two articles from 15 countries were included. Twelve articles explored drowning, while in 30 articles, drowning was reported as part of a wider study. The data sources were coronial, central registry, hospital record, sea rescue and self-generated data. Measures used to describe drowning were proportions and rates. There was a huge variation in the proportion and incidence rate of drowning reported by the studies included in the review. The potential risk factors for drowning included young age, male gender, ethnicity, alcohol, access to bodies of water, age and carrying capacity of the boat, weather and summer season. No study evaluated prevention strategies, however, strategies proposed were education, increased supervision and community awareness. *Conclusions:* There is a need to address the high rate of drowning in Africa. Good epidemiological studies across all African countries are needed to describe the patterns of drowning and understand risk factors. Further research is needed to investigate the risk factors and to evaluate prevention strategies.

## 1. Introduction

The World Health Organization (WHO) defines drowning as “the process of experiencing respiratory impairment from either immersion or submersion in liquid” [1]. Drowning is the third leading cause of unintentional injury related cause of mortality worldwide, accounting for 7% of all injury related deaths. It is a global under recognized and neglected public health burden that claims the lives of 320,000 people every year [2]. More than 90% of these deaths occur in low- and middle-income countries with inadequate prevention measures [3]. It is among the ten leading causes of deaths in children and young people in the world with children aged less than five years at increased risk [4]. Between 1990 and 2013, drowning rates declined by 52.2% globally [5], however, despite this decline, the highest rates of drowning were observed in Africa [3].

The African continent is unfortunately plagued with the world’s most dramatic public health crises with communicable diseases such as HIV/AIDS, malaria, and lower respiratory tract infections as leading causes of death in the region [6]. In addition, there is an increasing burden of non-communicable (hypertension, diabetes and heart) diseases and injuries [6]. The combined burden of both the communicable, non-communicable diseases and injuries have placed a strain on the already weak health systems in addition to struggling economies in the continent [6]. In spite of injuries been identified as a leading cause of death in Africa [4], this public health threat is yet to receive the desired attention, rather the management and prevention of communicable diseases is still a top priority [6].

Africa has recorded considerable success in reducing childhood deaths related to communicable diseases [6]. However, there is limited statistics and data on drowning related deaths which also contributes to infant mortality rates. Age is a leading risk factor for drowning and commonly occurs among children aged 1–4 years [4]. A recent incident that occurred in a West African country, was the case of a thirteen-month-old child who drowned in his parents’ indoor swimming pool [7]. This is a common occurrence in low- and middle-income countries (LMICs) and within the region [8]. Unfortunately, unlike high-income countries such as Australia and the United States of America (USA), drowning cases and deaths are under-reported in the African continent [4]. Other risk factors of drowning include, male gender, increased access to water, flooding disasters, commuting on water, lack of supervision and recreational drug use [4,9,10].

The knowledge of the epidemiology and risk factors of drowning aids the development and implementation of policies and strategies that reduce the incidence of drowning. Studies originating from high-income countries like Australia and USA suggest a range of primary and secondary prevention strategies to curb drowning deaths [11,12,13,14,15]. Recommendations proposed include increasing supervision, erecting pool fences, increasing public awareness and education through health promotion and public health advocacy [11,12,13,14,15]. However, these interventions may not be applicable in a region like Africa due to the diversity and variation in the epidemiologic, demographic and cultural factors [8].

Currently, there is no systematic review investigating drowning in Africa. Only three recent reviews on drowning in low- and middle-income countries, drowning in South Africa and Tanzania respectively have been published [8,16,17]. Therefore, it is imperative to understand the epidemiology, risk factors and current prevention strategies in Africa to direct policies for the prevention of drowning in Africa. Hence, the aim of this systematic review was to describe the epidemiology of drowning in Africa and to identify the risk factors and proposed and current strategies to prevent drowning.

## 2. Methods

### 2.1. Literature Search

The systematic review was conducted in accordance to the preferred reporting items for systematic review and meta-analysis guidelines (PRISMA) [18]. The PRISMA flow chart for the review is shown in Supplementary Figure S1. A literature search was conducted using Ovid Medline, Emcare, Cumulative Index to Nursing and Allied Health (CINAHL), PsycINFO, and Scopus for original research articles published in English from inception until the 30th of November 2018. The search was updated on the 1st April 2019. We included all articles focusing on drowning in Africa. There were slight variations in the search terms depending on the database. Search terms involved a combination of free text words and Medical Subject Headings (MeSH) terms. General search terms were “drown*” and “Africa”. The search strategy for Medline is shown in Supplementary Table S1. The study protocol was registered in PROSPERO with registration number CRD42019092758.

### 2.2. Eligibility Criteria

The studies included in this review are published original research reporting drowning in African countries. We applied no limits to the year of publication and included all age groups. In addition, we included studies that reported drowning as part of other injuries studies to capture all data from the region. Studies excluded were review articles, drowning because of suicide or homicide, non-fatal drowning or near drowning or hospitalization due to drowning or where fatal drowning could not be distinguished from non-fatal drowning.

### 2.3. Data Extraction

Faith O. Alele (F.O.A.) and Theophilus I. Emeto (T.I.E.) identified all included studies from the search strategy. Uncertainties about the included studies was discussed until consensus was reached. FOA and TIE extracted general and study specific characteristics from the included studies and Lauren Miller (L.M.) and Richard C. Franklin (R.C.F.) crosschecked the data.

### 2.4. Quality of Methods Assessment

The methodological quality of the included studies was assessed by FOA and TIE using the modified quality assessment tool for studies with diverse designs (QATSDD) critical appraisal tool [19]. The tool assesses the validity, reliability and generalizability of studies. The included studies were a mix of cross-sectional, descriptive and case-control studies and each study design were assessed using the appraisal tool. The tool was modified to exclude two items that were not applicable to the included studies. The excluded items comprised of statistical assessment of reliability and validity of measurement tool(s) (Quantitative only), fit between stated research question and format and content of data collection tool e.g., interview schedule (Qualitative), assessment of reliability of analytical process (Qualitative only) and evidence of user involvement in design. In the modified QATSDD tool each criterion was awarded a score of 0 to 3 with 0 = not at all, 1 = very slightly, 2 = moderately and 3 = complete. The scores of the criteria were summed up to assess the methodological quality of included studies with a maximum score of 36. For ease of interpretation, the scores were converted to percentages and were categorised as excellent (>80%), good (50–80%) and low (<50%) quality of evidence based on the overall score (Supplementary Table S2).

### 2.5. Data Synthesis

Drowning was reported exclusively or as part of a wider study such as injury studies. Approximately 28% (11) of the included studies reported drowning as unintentional. In studies where drowning was unspecified, we reported the drowning as intentional. Measures used to report drowning were proportions and incidence rates. The incidence rates and proportions of drowning were reported using frequency tables. The risk factors for drowning were identified in two articles, one of which only reported drowning as part of a wider study. Therefore, the risk factors identified were extrapolated to drowning. However, given the paucity of information on risk factors associated with drowning, we identified the potential risk factors based on previously identified factors documented in the literature [3,4,8] and based on the reported rates of the potential factors. A meta-analysis was not conducted due to the heterogeneity of the included studies.

## 3. Results

### 3.1. Epidemiology of Drowning in Africa

**Figure 1:**
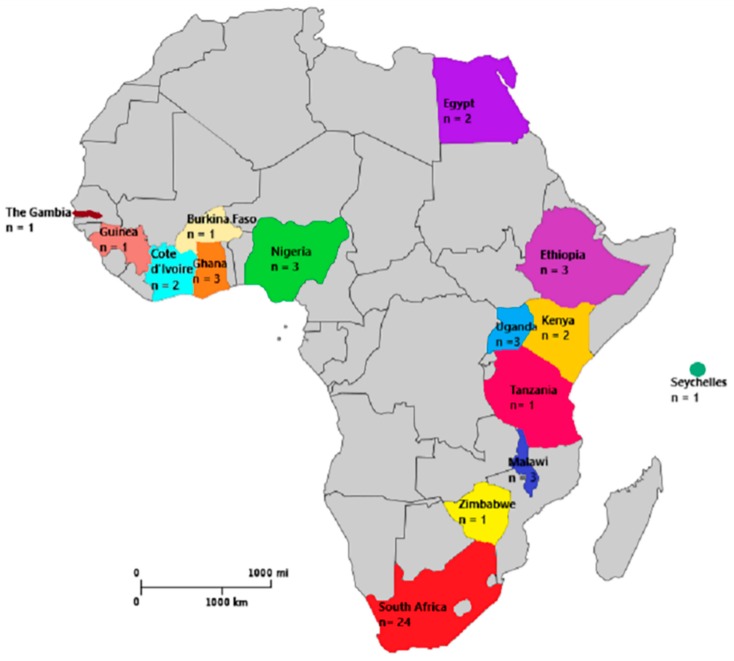
Map of Africa showing the countries and number of studies originating from each country was modified from Wikimedia Commons [63].

The included studies were conducted across 15 countries in Africa (Figure 1). The databases searches identified 345 articles, of which 42 articles were included in the review after screening for titles, abstracts and full text review (Supplementary Figure S1). Three (3) studies reported drowning in multiple sites (countries) [20,21,22]. Twenty-four (57%) of the articles originated from South Africa [20,21,22,23,24,25,26,27,28,29,30,31,32,33,34,35,36,37,38,39,40,41,42,43], three (7.1%) were from Ethiopia [20,44,45], Ghana [20,46,47], Malawi [20,48,49], Nigeria [50,51,52] and Uganda [53,54,55] respectively, while 2 (4.8%) studies were from Cote d’Ivoire [20,56], Kenya [20,57] and Egypt [21,22] respectively. One (2.4%) article each originated from Burkina Faso [20], Guinea [58], The Gambia [20], Tanzania [59], Seychelles [60] and Zimbabwe [61], See Figure 1 for location. The most commonly used data were surveillance data (46%) and death registers including hospital, police and coronial reports. Twelve (12) studies investigated and described drowning exclusively [21,22,23,24,25,26,27,30,43,50,54,60], while in 30 studies, drowning was reported as part of a wider study including studies investigation all cause of death and external causes of death [20,28,29,31,32,33,34,35,36,37,38,39,40,41,42,44,45,46,47,48,49,51,52,53,55,56,57,58,59,61]. Measures used to describe drowning were proportions and rates. Drowning rates in children were reported in thirteen (13) studies [21,28,30,31,33,35,37,38,39,42,46,49,52], while 29 (69%) studies described drowning rates of adults and children or adults alone [20,22,23,24,25,26,27,29,32,34,36,40,41,43,44,45,47,48,50,51,53,54,55,56,57,58,59,60,61]. The denominators used varied by country. In some studies, the estimated proportion or rate of drowning was based on the total population in the study area. By contrast, other studies reported the mortality rates for external causes and all cause of deaths. All deaths due to injuries, trauma and external causes were considered as mortality due to external causes using the ICD 11 classification [62].

### 3.2. Drowning Rates in Africa

In Table 1, twelve (12) investigated drowning exclusively in different regions of Africa [21,22,23,24,25,26,27,30,43,50,54,60]. However, there were variable methods of reporting drowning across the different studies. Among population-based studies, the proportion of drowning fatalities ranged from 0.019% to 1.2% [23,26]. In studies where all submersion events were reported, unintentional drowning accounted for 80% of drowning deaths in one study [50], while accidental drowning accounted for 10.7% of all submersion (near drowning and drowning) events in another study [30].

The incidence rates of drowning across the different studies ranged from a low of 0.33/100,000 population to a high of 502/100,000 population [21,22,24,25,27,43,54]. However, the denominators for each study varied. Two studies were conducted using the total population in the country as the denominator [21,22], five studies were conducted within specific cities and towns and total population of the cities were used as denominators [23,24,27,43,54], while one study investigated drowning across five cities [25].

Of the 30 studies reporting drowning as part of a wider study (Table 2), 25 studies reported drowning as part of external cause of death (injury) [20,28,29,31,32,33,34,35,36,40,41,42,44,45,46,47,48,49,51,52,53,55,57,58,59], while 5 studies investigated all causes of deaths and reported the rates of drowning [37,38,39,45,56]. The studies investigating external causes of deaths reported drowning deaths either as a proportion [28,29,31,32,33,34,40,41,42,44,46,47,49,51,52,55,57,61] or as a rate [20,35,36,48,53,58,59] of all external causes of deaths. The proportion of drowning deaths as part of external causes of death ranged from 0.2% to 75% [28,29,31,32,33,34,40,41,42,44,46,47,49,51,52,55,57,61] while the incidence rate ranged from 2.1/100,000 to 10.2/100,000 [35,36,48,58,59]. One ecological study which investigated external causes of deaths across eight (8) countries reported drowning rates ranging from 0/1000 person years to 0.48/1000 person years [20], while. Lett et al., reported that drowning rate account for 0.1/1000 people of all external cause of deaths [53]. In studies reporting drowning as a part of all causes of deaths, the proportion of drowning deaths ranged from 0.02% to 11% [37,38,39,45,56].

In terms of drowning rates by age demographics, the proportion of drowning among children and adolescents ranged from 2.5% to 19% [28,30,31,33,37,38,39,42,46,49,52], while the incidence of drowning ranged from 0.33 to 5.3 per 100,000 population [21,35]. The rates of drowning among adults ranged from 0.33 to 502 per 100,000 population [22,24,25,27,36,48,54,59] and from 0 to 0.07 per 1000 person years [20,53]. In addition, the proportion of drowning among adults ranged from 0.019% to 80% [23,26,29,32,34,40,41,44,45,50,51,55,56,57,58,60,61].

### 3.3. Potential Risk Factors

In Table 3, only two studies reported risk factors [48,58]. One study identified risk factors associated with drowning [58] and the other study reported risk factors of a wider study [58]. The identified risk factors were being a fisherman [58], having fishing as source of income [58], being male [58] and older age with the odds of drowning increasing with increasing age from 2.0 to 8.9 [58]. Potential risk factors were identified in 26 studies based on the rates of drowning reported and these potential factors include age, gender, ethnicity, alcohol, access to bodies of water, age of boat and carrying capacity of the boat, weather and summer season. In 19 studies, age was identified as a potential risk factor [24,25,26,27,28,30,31,32,33,37,40,42,46,47,55,57,58,59,60], with 13 studies reporting higher rates of drowning among children and adolescents [25,27,28,30,31,32,33,37,42,43,46,47,59]. In addition, males were reported to have higher rates of drowning compared to females in 15 studies [24,25,26,27,28,35,36,40,43,45,46,47,57,58,60]. Furthermore, six studies reported the rates of drowning for different races [24,26,28,31,35,42]. However, race as a potential risk factor varied between the included studies with three studies classifying the race by age groups [24,31,42]. The other three studies reported the rates of drowning for all age groups without grouping them [26,28,35]. Among studies reporting drowning rates for race by age groups, children aged <1 year–5 years of white African ethnicity were most vulnerable to drowning compared to other races. By contrast, drowning was more prevalent in adolescents and adults of black African ethnicity and Asian ethnicity [24,31,42}. Where no age classification was used, blacks of African ethnicity were reportedly had higher drowning rates in two studies [26,35], while one study reported a higher rate of drowning among whites of African ethnicity [28]. Other potential risk factors identified were alcohol [23,25,26], summer season [24,26,28,42,43], boat age and overloading of the boat [64], stormy weather [64] and access to bodies of water [24,25,26,31,43,48,54,55].

### 3.4. Prevention Strategies

Sixteen (16) studies proposed prevention strategies to reduce drowning rates in Africa [23,24,25,26,27,30,31,42,44,45,46,48,53,54,59,60]. These prevention strategies include increased supervision of children around bodies of water, aquatic education and training about basic life support measures, training about life skills in communities, community awareness and implementation of legislation to prevent drowning (Table 4). Using the hierarchy of controls [64], fourteen of the sixteen studies proposed administrative control/preventive measures, which included education/training on basic life support, legislative laws and increasing public awareness [23,25,27,30,31,42,44,45,46,48,53,54,59,60]. Two studies proposed engineering control measure that include building life safety facilities and the use of barriers and safety nets around swimming pools [24,26]. 

### 3.5. Methodological Quality Assessment

The QATSDD scores ranged from 53% to 100% (Supplementary Table S1). Thirty-three studies which scored above 80% [20,21,22,25,26,29,30,31,32,33,34,35,36,37,38,39,40,41,43,44,45,46,47,48,49,52,53,54,55,56,57,58,59], were categorised as having excellent methodological quality, and included details about sampling, data analysis, strengths and limitations of the study. The other studies were considered to be of good methodological quality [23,24,27,28,42,50,51,60,61] and no study scored below 50%. The included studies utilised retrospective data or collected information retrospectively from participants. Inaccuracy and incompleteness of the data may be associated with the use of retrospective data. In addition, misclassification bias may have been introduced into the studies that used retrospective data. Furthermore, depending on the participants, recall bias may have been introduced into some of the studies. These biases could have underestimated or overestimated the burden of drowning in Africa.

## 4. Discussion

Drowning is a significant public health burden in Africa and the findings of this systematic review suggest that there is a huge variation in drowning mortality across Africa. The highest proportion of drowning (approximately 80%) was reported in Nigeria [50], while the highest rate reported (502/100,000 population) was observed in Uganda [54]. Although only two studies identified risk factors which includes being a fisherman, and older age [48,58]; we identified potential risk factors based on previous evidence [3]. The limited evidence suggests that male gender and young people are at higher risk for drowning especially children and adolescents. In addition, other potential risk factors identified were being of black African ethnicity, alcohol use, access to bodies of water, age of boat and carrying capacity of the boat, weather and summer season. This systematic review has highlighted the need for more data on drowning prevalence, together with good epidemiological studies across all African countries to describe the patterns of drowning and understand risk factors to guide prevention initiatives.

Due to the limited data available, quantifying the prevalence of drowning in Africa was challenging. This finding was echoed in a study of drowning in low- and middle-income countries by Tyler et al. who reported that inconsistences in data collection for drowning poses as a challenge for data synthesis [8]. Although the estimated rates and proportions may be considered high, in majority of the studies, drowning was reported as part of a wider injury study. Of the 54 countries in Africa, only 15 countries had some published data on drowning with the majority (57%) of the literature originating from South Africa. In many African countries, cases go unreported and the lack of an injury surveillance system as seen in many LMIC also contributes to the limited data [65]. This is consistent with the findings of two recent systematic reviews describing the burden of drowning in South Africa and Tanzania [16,17]. Saunders et al. and Sarrassat et al., reported that strengthening the existing surveillance systems or establishing new ones are needed for consistent and detailed drowning surveillance [16,17]. In addition, as many of the drowning cases result in death at the time of the event, only a small proportion present at the hospital or medical facilities [66]. Both the lack of the injury surveillance system and underreporting of drowning cases prevent accurate documentation of drowning mortality in health records. According to WHO, approximately 90% of global drowning deaths occur in LMICs. Africa as a region had an estimated 73,635 drowning deaths in 2016 which accounted for approximately 23% of the total drowning deaths globally [67]. However, data collection in the region is limited, and hence the statistics from Africa underrepresents the true burden of drowning in the region [4].

Many of the potential risk factors associated with drowning identified in this review are similar to those reported in previous systematic reviews. Drowning occurred more frequently in males between the ages of 0 to 15 years. Specifically, highest drowning occurrences were found in the 0–5-year age group. In LMICs, drowning rates among children were higher among children aged 1–4 years, followed by children aged 5–9 years with males being twice as likely as females to drown [8]. In addition, younger children were found to drown in private pools or baths, whereas older children were found to drown in public swimming pools, rivers, dams or in the ocean. Given that a majority of the studies originated from South Africa, drowning in swimming pools occurred more in white African children, whereas drowning in dams and rivers were found to occur in older black African children [24,31]. Although it is not evident that socioeconomic status is a potential risk factor in this review, children from low-income households may not have access to private swimming pools and are more likely to access natural bodies of water around the house as reported in other LMICs [8]. Evidence suggests that a lack of child supervision and the lack of safety barriers has been associated with high drowning rates among children [66]. Furthermore, access to other water bodies using boats or through fishing, depending on occupational or recreational purposes was also considered as a potential risk factor [48,54]. Specifically, children and adults from fishing households, are more likely to access these types of water bodies daily, requiring significant surveillance and awareness strategies for children in such settings. Other potential risk factors such as alcohol consumption has been shown to be associated with drowning especially among adolescents and adult [68]. As blood alcohol concentration level rises, judgement, balance and vision may be impaired, increasing the risk of drowning. Binge drinking is common in some African countries and has been reported to be associated with drowning among adult men [23,24,25,26,50]. This calls for increased awareness of the risk of alcohol consumption in conjunction with swimming.

The prevention strategies proposed by sixteen studies includes focusing on pool safety such as restricting access to private pools for young children, education and training at schools on life skills, increasing public awareness through media campaigns, and the implementation of water safety legislation, community awareness, improved supervision of children around water bodies, building lifesaving facilities and enforcement of boat construction and maintenance regulations. Using the hierarchy of controls which is a system used to minimize or eliminate hazards [64], only two studies proposed engineering controls as a way of preventing drowning [24,26]. All other studies proposed prevention strategies that require administrative controls which is the least effective way to prevent drowning [23,25,27,30,31,42,44,45,46,48,53,54,59,60]. However, prevention interventions and methods may not be consistent between countries due to the diversity and variation in their epidemiology, demographic and cultural characteristics [8]. There is no simple solution to addressing the burden of drowning in all countries, therefore strategies would have to be designed specifically for each country, keeping in mind the cultural, economic and social structures.

### 4.1. Implications for Policy and Future Research

The findings of this review suggest that there is limited evidence and data on the burden of drowning in Africa. The 2017 Global Burden of Disease (GBD) using statistical models estimated that drowning contributed approximately 0.53% of the total deaths in Africa as a region. Using the GBD to obtain country specific estimates for the countries included in the review showed an average drowning mortality ranging from 1.63 per 100,000 population to 5.73 per 100,000 population [69]. However, existing data on drowning in many countries in Africa are scarce. Therefore, establishing specific databases about injuries like drowning for surveillance and data collection would aid in development of policies and prevention strategies across the different countries. Evidence from research conducted in high income countries like Australia, Canada and New Zealand suggest that robust high-quality data and better data collection system would enable the creation of targeted and effective drowning prevention interventions [70]. Developing the databases will enable cross-country comparison which allows for identification of similarities and improvements in data collection. However, establishing the databases may be challenging for some African countries, especially if drowning is not among the national health priorities. In addition, there was little exploration of the risk factors associated with drowning, highlighting a gap in the literature. Good epidemiological studies are needed to identify the risk factors and evaluate the proposed prevention strategies for drowning in Africa. Furthermore, future research should focus on the intent for drowning in Africa, which would help to inform policies and prevention interventions. A recent article on intentional drowning reported an increasing rate of intentional drowning and proposed a multidisciplinary collaboration public health and other services including mental health, education and drowning prevention organisations to prevent intentional drowning [71].

### 4.2. Strengths and Limitations

To our knowledge, this is the first systematic review that describes the epidemiology, risk factors and prevention strategies of drowning in Africa. However, comparing mortality data across the countries within Africa needs to be undertaken with caution given the different measures used to analyse the burden of drowning. Some studies were population-based studies, while other studies reported drowning as a part of a wider study (such as external causes of deaths or all causes of death). An example of the latter is the study by Seleye-Fubara et al. which reported that unintentional drowning accounted for approximately 80% of all drowning deaths [50]. In addition, the completeness and reliability of the data in each country varied with some studies using the national mortality surveillance statistics, while other studies relied on hospital-based data, mortuary-based data and demographic surveillance data. The variability in the sources of data may account for the variable rates of drowning reported in the review.

Other limitations include reviewing only articles published in peer-reviewed journals. We may have missed other high-quality studies that are published in non-peer reviewed journals. In addition, we excluded non-English articles, there is a possibility that we may have missed articles from Africa published in other languages. Furthermore, the majority of studies were from South Africa, it is conspicuous that there is a paucity of data from many countries in Africa. It is uncertain whether such strategies, if implemented, can be generalizable to other African countries besides South Africa.

## 5. Conclusions

There is a need to address the high rate of drowning in Africa. It is imperative that governments across the nations of Africa establish good injury surveillance systems to accurately understand the burden of drowning to inform approaches for drowning prevention. Good epidemiological studies across all African countries are needed to describe the patterns of drowning and understand risk factors. Further research is needed to investigate the risk factors and to evaluate prevention strategies.

## Figures and Tables

**Table 1:** Summary of studies exclusively reporting drowning in Africa.

Authors, Reference, Year	Country	Study Design	Year	Study Population	Rates and Proportion of Drowning
Davis and Smith, 1982 [23]	South Africa (Cape Town)	Descriptive cross-sectional study	1979–1981 (3 years)	1,500,000 people (population in Cape Town)	285 (0.019%) drowning deaths ^§^
Grainger 1985 [60]	Seychelles	Descriptive cross-sectional study	1959–1978 (20 years)	119 drowning deaths	5.95 ± 2.2 drownings per year (mean drowning rate)
Davis and Smith 1985 [24]	South Africa (Cape Town)	Descriptive cross-sectional study	1980–1983 (4 years)	1,600,000 people (population in Cape town)	Male: 38.7/100,000
					Female: 8.3/100,000
Meel BL, 2008 [27]	South Africa (Mthatha)	Descriptive cross-sectional study	1993–2004 (12 years)	400,000 people (population in Mthatha)	Mean drowning rate: 7.1/100,000
Seleye-Fubara et al., 2012 [50]	Nigeria (Niger-delta region)	Descriptive cross-sectional study	1998–2009 (12 years)	85 drowning deaths	80% were unintentional drowning
Donson and Nickerk, 2013 [25]	South Africa	Descriptive cross-sectional study	2001–2005 (5 years)	Total population in five cities (Johannesburg, Durban, Cape Town, Port Elizabeth and Pretoria)	2.1/100,000
Joanknecht et al., 2015 [30]	South Africa: Cape Town	Descriptive cross-sectional study	2007–2013 (6 years)	75 children admitted for a submersion incident (near drowning and drowning)	10.7% of the study population drowned
Lin et al., 2015 [22]	Egypt South Africa	Ecological study	2009–2011 (3 years) 2007–2009 (3 years)	Entire population in the country Entire population in the country	1.5/100,000 2.5/100,000
Morris et al., 2016 [26]	South Africa (Pretoria)	Descriptive cross-sectional study	2002–2011	23,050 registered deaths 278 deaths due to external causes	1.2% (278) of the deaths were due to drowning
Kobusingye et al., 2017 [54] ^‡^	Uganda (Buikwe; Kampala; Mukono; Wakiso)	Mixed methods: Quantitative—Cross-sectional ^ǂ^	Not stated	2804 people (population in the community)	502/100,000
Wu et al., 2017 [21]	Egypt South Africa	Ecological study	2000 and 2013	WHO world standard population WHO world standard population	Unintentional drowning rate 2000: 3.89/100,000 2013: 2.93/100,000 2000: 0.33/100,000 2013: 3.38/100,000
Saunders et al., 2018 [43]	South Africa (Western Cape)	Descriptive cross-sectional	2010–2016 (6 years)	Total population in Western Cape (not stated by the authors)	3.2/100,000

^§^ Drowning proportions was calculated using data provided in the article. ^‡^ For the purpose of the review, only the quantitative aspect of the study was included in the review.

**Table 2:** Studies describing drowning as part of other studies (including external causes and all causes) in Africa.

Authors, Reference, Year	Country	Study Design	Year	Study Population	Rates and Proportion of Drowning
Chitiyo 1974 [61]	Zimbabwe (Bulawayo area)	Descriptive cross-sectional study	1972	188 adult deaths (external causes)	11.17% (21) drowning deaths
Knobel et al., 1984 [28]	South Africa	Descriptive cross-sectional study	1966–1981 (15 years)	3248 children < 15 years (external causes)	356 drowning deaths (11%)
Kibel et al., 1990 [31]	South Africa	Descriptive cross-sectional study	1981–1985 (5 years)	14,118 children under 15 years of age (deaths due to external causes)	19% of all injury related deaths
Flisher et al., 1992 [42]	South Africa	Descriptive cross-sectional study	1984–1986 (3 years)	9288 adolescent deaths due to external causes	10.8% of all deaths due to external causes
Lerer et al., 1997 [32]	South Africa (Cape Town)	Descriptive cross-sectional	1994 (1 year)	3690 deaths due to external causes	2.6% (96) of all non-natural mortality was due to drowning
Kobusingye et al., 2001 [55]	Uganda (Mukono district)	Descriptive cross-sectional	1993–1998 (5 years)	34 fatal injuries (external causes)	27% (9) of fatal injuries were due to drowning
Moshiro et al., 2001 [59]	Tanzania	Descriptive cross-sectional study	1992–1998 (6 years)	64,756 persons in Dr es Salaam	Overall drowning incidence not stated
				146,359 (population in Hai)	Female drowning rates/100,000
				103,053 (population in Morogoro)	Dar es Salaam: 4.7
				1478 deaths due to injuries (external causes) all age groups	Hai District: 5.5
					Morogoro: 5.1
					Male drowning rates/ 100,000
					Dar es Salaam: 9.2
					Hai District: 10.2
					Morogoro: 7.9
Lett et al., 2006 [53]	Uganda (Gulu district)	Descriptive cross-sectional study	1994–1999 (5years)	8595 people397 deaths due to external causes	0.1/1000 people were due to drowning
Osime et al., 2007 [51]	Nigeria (Benin City)	Descriptive cross-sectional study	2001–2004 (4 years)	5446 trauma related deaths (external causes)	Drowning accounted for 0.8% of all trauma related deaths
Burrows et al., 2010 [35]	South Africa	Descriptive cross-sectional study	2001–2003 (2 years)	3,301,190 children aged 0–14 years 2923 injury related deaths (external causes) of children aged 0–14 years	Female: 2.1/100,000
					Male: 5.3/100,000
Ohene et al., 2010 [46]	Ghana (Accra)	Descriptive cross-sectional	2001–203 (2 years)	151 injury related deaths (external causes) among adolescents aged 10–19 years	38% of deaths were due to drowning
Garrib et al., 2011 [40]	South Africa	Analytical cross-sectional	2000–2007 (7 years)	133,483 people 1022 injury related deaths (external causes)	3.3% due to drowning
Mendes et al., 2011 [29]	South Africa (Johannesburg)	Descriptive cross-sectional study	2006–2009 (4 years)	1760 unintentional injuries (external causes)	0.34% of the deaths were due to drowning ^§^
Mamady et al., 2012 [58]	Guinea	Analytical cross-sectional study	2007	9,710,144 (total population) 7066 fatal injuries (external causes)	4.4/100,000
Odhiambo et al., 2013 [57]	Kenya	Analytical cross-sectional	2003–2008 (5 years)	220,000 people (total population) 11,147 adult deaths due to trauma (external causes)	0.2% (23) deaths were due to drowning
Weldearegawi et al., 2013 [45]	Ethiopia (Kilite Awlaelo surveillance site)	Descriptive cross-sectional study	2009–2011 (3 years)	409 deaths (all causes)	4.6% of all deaths were due to drowning
Streatfield et al., 2014 [20]	Burkina Faso Cote d’Ivoire Ethiopia The Gambia Ghana Kenya Malawi South Africa	Ecological study	2000-2012 (3 years)	111,910 deaths/ 12,204,043 person-years across Africa and Asia due to external causes	Rates/1000 person years Burkina Faso (Nouna): 0.00 Burkina Faso (Ouagadougou): 0.20 Cote d’Ivoire (Taabo): 0.14 Ethiopia (Kilite Awlaelo): 0.13 The Gambia (Farafenni): 0.11 Ghana (Dodowa): 0.28 Ghana (Navrongo): 0.48 Kenya (Kilifi): 0.18 Kenya (Kisumu): 0.22 Kenya (Nairobi): 0.18 Malawi (Karonga): 0.19 South Africa (Africa Centre): 0.19 South Africa (Agincourt): 0.07
Chasimpha et al., 2015 [48]	Malawi (Karonga district)	Nested case-control	2002 2012	59,947 people (children and adults) in Karonga districtDeaths due to external causes	Unintentional drowning rate: 8.6/100,000
Kone et al., 2015 [56]	Cote d’Ivoire	Descriptive cross-sectional study	2009–2011 (3 years)	39,422 people (total population)712 deaths (all causes)	Unintentional drowning rates
					Male *
					5–14: 0.1%
					15–49: 0.3%
					Female *
					5–14 years: 0.02%
Matzopoulos et al., 2015 [36]	South Africa	Descriptive cross-sectional study	2009	52,493 injury related deaths (external causes)	Unintentional drowning3.3/100,000
Olatunya et al., 2015 [52]	Nigeria (Ekiti State)	Descriptive cross-sectional study	2012–2014 (2 years)	5264 children admitted for injury related incidents (external causes)	Drowning accounted for 4.54% of all injuries
Pretorius and Niekerk, 2015 [33]	South Africa: Guateng	Descriptive cross-sectional study	2008–2011 (2 years)	Total population in Gauteng 5404 fatal injuries (external causes) in children aged 0–19 years	8.9% of all fatal injuries were due to drowning
Groenewald et al., 2016 [38]	South Africa: Western Cape	Descriptive cross-sectional study	2011	2412 deaths (all causes) of children under 5 years of age	Drowning accounted for 2.8% of all deaths
Mathews et al., 2016 [37]	South Africa: Western Cape and KwaZulu-Natal	Descriptive cross-sectional study	2014	711 child deaths (all causes)	Drowning deaths accounted for 2.5% of all deaths
Reid et al., 2016 [39]	South Africa: Western Cape	Descriptive cross-sectional study	2011	180,814 children under 5 years of age (total population) 1051 under-5 deaths (all causes)	11% of all deaths were due to drowning
Meel BL, 2017 [34]	South Africa	Descriptive epidemiology	1996–2015 (20 years)	24, 693 deaths due to unnatural (external) causes	5.1% of unnatural deaths were due to drowning
Purcell et al., 2017 [49]	Malawi: Lilongwe	Descriptive cross-sectional study	2008–2013 (6 years)	30,462 children with traumatic injuries 343 deaths due to external causes	11.4% of the deaths were due to drowning
Erasmus et al., 2018 [41]	South Africa	Descriptive cross-sectional study	2010–2014 (5 years)	184 injuries related (external causes) deaths over the time period	75% (138) of the deaths were due to drowning
Gelaye et al., 2018 [44]	Ethiopia	Descriptive cross-sectional study	2009–2013 (5 years)	623 injury related deaths (external causes)	21.8% (136) deaths were due to drowning
Ossei et al., 2019 [47]	Ghana	Descriptive cross-sectional study	2008–2016 (8 years)	1470 unnatural deaths (external causes)	7.14% of the deaths were due to drowning

^§^ Drowning proportions were calculated using data provided in the articles. * Other age groups reported no drowning deaths.

**Table 3:** Studies discussing potential risk factors for drowning among all age groups in Africa.

Authors, Reference, Year	Country	Study Population	Proportions or Rates of Potential Risk Factors			Potential Risk Factors/Risk Factors Identified	
Davis and Smith, 1982 [33]	South Africa (Cape Town)	1,500,000 people				Alcohol	
Knobel et al., 1984 [28]	South Africa	3248 children < 15 years	Race			Race: whites	
			Coloured		10.7%		
			**White**		**16.1%**		
			Black		7.9%		
			Gender			Male	
			**Male**		**11.7%**		
			Female		9.7%		
			Age				
			<1 year		6.9%	Age: 6–14	
			1–5 years		12.8%		
			**6–14 years**		**20.3%**	Summer season	
						Weekends	
Davis and Smith 1985 [24]	South Africa (Cape Town)	1,600,000 people	Race			Race: Black race for adults >30 years	
			**Black race**		**32.3/100,000**	White race for children 0–5 years	
			Colored		24.2/100,000	Male	
			White		13.4/100,000		
			Gender				
			**Male**		**38.7/100,000**		
			Female		8.3/100,000		
			Age			Age: 21–30	
			0–5 years		13.3%		
			6–10 years		5.8%		
			11–15 years		4.9%		
			16–20 years		10.98%		
			**21–30 years**		**25.14%**	Summer season	
			31–40 years		15.61%	Swimming pools	
			>40 years		24.28%	Alcohol	
Grainger 1985 [60]	Seychelles	119 drowning deaths	Age			Age: 40–49 years	
			0–9 years		6.72%	Epilepsy	
			10–19 years		13.44%	Head injury	
			20–29 years		16.8%	Time of day: 12–2 pm	
			30–39 years		18.5%		
			**40–49 years**		**21.8%**		
			50–59 years		12.6%		
			60–69 years		5.88%		
			70+ years		4.2%		
			Gender				
			**Male**		**109 deaths**	Male	
			Female		10 deaths		
Kibel et al., 1990 [31]	South Africa	14,118 children under 15 years of age (injuries related death)	Age			Age: 1–4 years	
			<1year		7.4%		
			**1–4 years**		**23.0%**		
			5–14 years		20.1%		
			Race		<1 year	White race for children <1 year to 4 years	
			Blacks		6.7%	Black race for children aged 5–14 years	
			**Whites**		**9.5%**	Site:	
			Coloured		8.3%	Swimming pools for white children	
			Asians		6.4%	Dams and rivers for older black children	
					1–4 years		
			Blacks		18.8%		
			**Whites**		**42.7%**		
			Coloured		22.1%		
			Asians		9.4%		
					5–14 years		
			**Blacks**		**21.9%**		
			Whites		12.7%		
			Coloured		21.2%		
			Asians		9.4%		
Flisher et al., 1992 [42]	South Africa	9288 adolescent deaths due to external causes	Race		10–14 years	Age: 10–14 years	
			Whites		6.3%	Black race for adolescents 10–14 years old	
			Coloured		25.2%	Asian race for adolescents 15–19 years old	
			Asians		12.7%	Summer season	
			**Black**		**24.2%**		
			Race		15–19 years		
			Whites		4.2%		
			Coloured		9.7%		
			**Asians**		**12.5%**		
			Black		6.3%		
Lerer et al., 1997 [32]	South Africa (Cape Town)	3690 non-natural mortality	Age		Number of drowning deaths	Age: 0–14 years	
			**0–14 years:**		**35**		
			15–24 years:		10		
			25–34 years		16		
			35–44 years		16		
			45–54 years		12		
			55–64 years		3		
			65–74 years		1		
			75+ years		3		
Kobusingye et al., 2001 [55]	Uganda (Mukono district)	34 fatal injuries	Age		% of drowning	Age: 10–39 years	
			<10 years		0%	Extensive water surface	
			10–19 years		18%		
			20–29 years		18%		
			30–39 years		18%		
			40–49 years		0%		
			>50 years		0%		
Moshiro et al., 2001 [59]	Tanzania	1478 deaths due to injuries all age groups	Female gender/100,000 population			Females;	
				Dar es Salaam	Hai District	Morogoro	0–4 years (across the three districts)
			**0–4 years**	**7.0**	**17.1**	**6.9**	
			5–14 years	2.2	5.0	6.0	Males:
			15–59 years	5.2	3.0	4.2	Dar es Salaam: 15–59 years
			60+ years		2.8	4.6	Hai District: 0–4 years
						Morogoro: 60 years and above	
			Male gender/100,000 population				
				Dar es Salaam	Hai District	Morogoro	
			0–4 years	3.4	**12.3**	12.1	
			5–14 years	4.9	9.0	3.6	
			15–59 years	**12.4**	10.6	7.9	
			60+ years		8.5	**16.1**	
Meel BL, 2008 [27]	South Africa (Mthatha)	405 drowning deaths		Male	Female	Male	
			**1–10 years**	**23.4%**	**6.9%**	Age: 1–20 years	
			**11–20 years**	**15.2%**	**9.2%**		
			21–30 years	9.8%	4.7%		
			31–40 years	11.3%	2.8%		
			41–50 years	5.0%	1.3%		
			51–60 years	3.8%	0.6%		
			61+ years	3.1%	2.5%		
Burrows et al., 2010 [35]	South Africa	2923 injury deaths of children aged 0–14 years			Drowning rate per 100,000		
			Gender				
			Female		2.1	Buffalo City	
			**Male**		**5.3**		
						Male	
			Population group				
			Asian		0.9		
			White		4.3		
			Coloured		2.0		
			**African**		**4.3**	African	
			City				
			Tshwane		2.9		
			Cape Town		2.2		
			Johannesburg		4.2		
			eThekwini		3.8		
			Nelson Mandela		3.7		
			**Buffalo City**		**9.2**		
Ohene et al., 2010 [46]	Ghana (Accra)	151 injury related deaths among adolescents aged 10–19 years	Gender			Male	
			Female		25%		
			**Male**		**44%**		
			Age				
			**10–14 years**		**46%**	Age: 10–14 years	
			15–19 years		33%		
Garrib et al., 2011 [40]	South Africa	1022 injury related deaths	Age			Children aged 0–15 years	
			**0–15 years**		**65%**		
			>15 years		35%		
			Gender (rate/100,000 person years)				
			**Male**		**6.2**	Male	
			Female		3.4		
Mamady et al., 2012 [58]	Guinea	7066 fatal injuries				Female	Ref
						**Male**	**OR 2.8; 95% CI (2.3–3.5)**
						0–4 years	Ref
						**5–14 years**	**OR 2.0; 95% CI (1.1–3.5)**
						**15–24 years**	**OR 8.9; 95% CI (5.3–15.0)**
						**25–64 years**	**OR 7.0; 95% CI (4.2–11.7)**
						**65+ years**	**OR 7.9; 95% CI (4.4–14.3)**
Seleye-Fubara et al., 2012 [50]	Nigeria (Niger-delta region)	85 drowning deaths				Alcohol	
						Hard drugs	
						Epilepsy	
Donson and Nickerk, 2013 [25]	South Africa	1648 drowning deaths	Age (rate/100,000)				
			**0–4 years**		**6.3**	0–4-year age group	
			5–14 years		2.2		
			15–29 years		1.7	Swimming pools	
			30–44 years		1.8		
			45–59 years		1.4	Alcohol use	
			60+ years		1.2		
			Gender (rate/100,000)			December	
			**Male**		**3.4**		
			Female		0.9	Male	
Odhiambo et al., 2013 [57]	Kenya	11,147 adult deaths due to trauma	Gender				
			Female		13%	Male	
			**Male**		**87%**		
			Age				
			**<40 years**		**83%**	Young adults (<40 years of age)	
			>40 years		17%		
Weldearegawi et al., 2013 [45]	Ethiopia (Kilite Awlaelo surveillance site)	409 deceased	Gender			Male	
			Female		1.7%		
			**Male**		**2.9%**		
Chasimpha et al., 2015 [48]	Malawi (Karonga district)	59,947 people (children and adults) in Karonga district				Children from fishing households	OR 3.07; 95% CI (1.03–9.10) ^‡^
						Adult male with fishing as a source of income	OR 2.45; 95% CI (1.17–5.14) ^‡^
						Adult males who are fishermen	OR 2.92; 95% CI (1.42–5.98) ^‡^
						Adult females who have other occupations	OR 4.04; 95% CI (1.22–13.4) ^‡^
Joanknecht et al., 2015 [30]	South Africa (Cape Town)	75 children admitted for a submersion incident				Public pools and the ocean for children older than 5 years of age	
						Private pools, baths and buckets for children less than 5 years	
Matzopoulos et al., 2015 [36]	South Africa	52,493 injury related deaths	Gender (rate/100,000 population)			Male	
			Female		1.2		
			**Male**		**5.7**		
Pretorius and Niekerk, 2015 [33]	South Africa (Guateng)	5404 fatal injuries in children aged 0–19 years	Age				
			0–1 year		9.4%	Age: 2–3 years	
13.4%			**2–3 years**		**16.8%**		
			4–6 years		13..4%		
			7–12 years		13.1%		
			13–19 years		3.0%		
Mathews et al., 2016 [37]	South Africa (Western Cape and KwaZulu-Natal)	711 child deaths	Age				
			<1 year		1.1%	Age: 1–4 years	
			**1–4 years**		**5.8%**		
			5–14 years		5.0%		
			15–17 years		1.9%		
Morris et al., 2016 [26]	South Africa (Pretoria)	346 deaths due to external causes	Gender				
			Female		21%	Male	
			**Male**		**79%**		
			Race				
			**Black**		**71%**	Black race	
			White		24%		
			Coloured		4%		
			Asian		1%		
			Age			Age >18 years	
			<1 year		15%		
			1–2 years		19%	Summer months (December to February)	
			2–13 years		18%	Alcohol	
			13–18 years		3%	Swimming pool	
			**>18 years**		**45%**		
Kobusingye et al., 2017 [54]	Uganda (Buikwe; Kampala; Mukono; Wakiso)	2804 people (population in the community)				Access to water bodies (for transportation or fishing)	
						Overloading	
						Stormy weather	
						Old age of boats	
Meel BL, 2017 [34]	South Africa	24,693 deaths due to unnatural causes	Gender			Female	
			**Female**		**6.07%**		
			Male		4.8%		
Gelaye et al., 2018 [44]	Ethiopia	623 injury related deaths	Gender			Female	
			**Female**		**22%**	No formal education (illiterates)	
			Male		21.1%		
Saunders et al., 2018 [43]	South Africa (Western Cape)	1391 drowning deaths	Age (rate/100,000 population)			Age: 0–19 years	
			Children (0–19 years)		3.8		
			Adults (20+ years)		3.0		
			Gender (rate /100,000 population)				
			Female		1.2	Male	
			**Male**		**5.3**	Access to large open bodies of water	
						Summer season (December, January, February)	
Ossei et al., 2019 [47]	Ghana	1470 unnatural deaths	Age			Age: 0–9 years	
			**≤9 years**		**40%**		
			10–19 years		17.14%		
			20–29 years		17.14%		
			30–39 years		10.48%		
			40–49 years		6.67%		
			50–59 years		3.81%		
			60–69 years		2.86%		
			≥70 years		1.90%		
			Gender			Male	
			Female		22.9%		
			**Male**		**77.1%**		

^‡^ Risk factors for external death, which also applies to drowning.

**Table 4:** Studies discussing proposed prevention strategies for drowning among all age groups in Africa.

Authors, Reference, Year	Country	Study Population	Prevention Strategy	Hierarchy of Controls
Davis and Smith, 1982 [23]	South Africa (Cape Town)	1,500,000 people	Increase public awareness	Administrative control
			Media campaign to reduce drinking in combination with aquatic activities	
Davis and Smith 1985 [24]	South Africa (Cape Town)	1,600,000 people	Live saving facilities	Engineering control
			Improved adult supervision of children	Administrative control
Grainger 1985 [60]	Seychelles	165 deaths due to accidents	Primary health education proposed	
		119 drowning deaths		
Kibel et al., 1990 [31]	South Africa	14,118 children under 15 years of age	Increase public awareness	Administrative control
			Safety legislation to reduce environment hazards	Administrative control
Flisher et al., 1992 [42]	South Africa	9288 adolescent deaths	Media efforts/ intervention to prevent drowning	Administrative control
Moshiro et al., 2001 [59]	Tanzania	1478 deaths due to injuries	Education	Administrative control
Lett et al., 2006 [53]	Uganda (Gulu district)	397 deaths due to injuries associated with war	Formal monitoring by international bodies with no political or economic interest in the conflict.	Administrative control
Meel BL, 2008 [27]	South Africa (Mthatha)	405 drowning deaths	Education and training at schools about life skills	Administrative control
Ohene et al., 2010 [46]	Ghana (Accra)	151 deaths among adolescents aged 10–19 years	Aquatic safety education	Administrative control
			Supervision near recreational water bodies	Administrative control
Donson and Nickerk, 2013 [25]	South Africa	1648 drowning deaths	Public aquatic safety education	Administrative control
			Implementation of evidence-led safety measures	Administrative control
			Water safety legislation	Administrative control
Weldearegawi et al., 2013 [45]	Ethiopia (Kilite Awlaelo surveillance site)	409 deceased	Integrating occupational and safety education with existing health programme to reduce mortalities associated with accidents	Administrative control
Chasimpha et al., 2015 [48]	Malawi (Karonga district)	59,947 people (children and adults)	Improved supervision of children around bodies of water	Administrative control
Joanknecht et al., 2015 [30]	South Africa (Cape Town)	75 children admitted for a submersion incident	Community based education and prevention programs focusing on restricting access to private pools for young children	Administrative control
Morris et al., 2016 [26]	South Africa (Pretoria)	346 deaths due to external causes	Public education regarding basic life support measures and dangers of alcohol consumption and swimming	Administrative control
			Use of safety nets/barriers	Engineering control
Kobusingye et al., 2017 [54]	Uganda (Buikwe; Kampala; Mukono; Wakiso)	2804 people	Enforce boat construction and maintenance regulations	Administrative control
			Loading limits	Administrative control
			Boat crew training	Administrative control
			Use of weather forecast	Administrative control
Gelaye et al., 2018 [44]	Ethiopia	623 injury related deaths	Community awareness	Administrative control

## Supplementary Materials

The following are available online at https://www.mdpi.com/1010-660X/55/10/637/s1. Table S1: Medline search strategy; Table S2: Quality assessment of included studies using the quality assessment tool for studies with diverse designs (QATSDD); Figure S1: Schematic of study inclusion.

## References

[1] van Beeck E.F., Branche C., Szpilman D., Modell J.H., Bierens J.J. (2005). A new definition of drowning: Towards documentation and prevention of a global public health problem. Bull. WHO.

[2] World Health Organization (WHO) Drowning: 2019. https://www.who.int/violence_injury_prevention/drowning/en/.

[3] World Health Organization (WHO) Drowning: 2018. https://www.who.int/en/news-room/fact-sheets/detail/drowning.

[4] World Health Organization (2014). Global Report on Drowning: Preventing a Leading Killer.

[5] Haagsma J.A., Graetz N., Bolliger I., Naghavi M., Higashi H., Mullany E.C., Abera S.F., Abraham J.P., Adofo K., Alsharif U. (2016). The global burden of injury: Incidence, mortality, disability-adjusted life years and time trends from the Global Burden of Disease study 2013. Inj. Prev..

[6] World Health Organization (WHO) (2014). The African Regional Health Report: The Health of the People.

[7] Agbo N. (2018). D’Banj Loses Son. The Guardian.

[8] Tyler M.D., Richards D.B., Reske-Nielsen C., Saghafi O., Morse E.A., Carey R., Jacquet G.A. (2017). The epidemiology of drowning in low- and middle-income countries: A systematic review. BMC Public Health.

[9] Borse N., Sleet D.A. (2009). CDC Childhood Injury Report: Patterns of Unintentional Injuries Among 0- to 19-Year Olds in the United States, 2000–2006. Fam. Community Health.

[10] Modell J.H. (2010). Prevention of needless deaths from drowning. South. Med. J..

[11] Moran K., Quan L., Franklin R., Bennett E. (2011). Where the evidence and expert opinion meet: A review of open-water recreational safety messages. Int. J. Aquat. Res. Educ..

[12] Quan L., Bennett E.E., Branche C.M., Doll L., Bonzo S., Sleet D., Mercy J., Haas E.N. (2008). Interventions to prevent drowning. Handbook of Injury and Violence Prevention.

[13] Szpilman D., Bierens J.J., Handley A.J., Orlowski J.P. (2012). Drowning. N. Engl. J. Med..

[14] Ramos W., Beale A., Chambers P., Dalke S., Fielding R., Kublick L., Langendorfer S.J., Lees T., Quan L., Wernicki P. (2015). Primary and secondary drowning interventions: The American Red Cross circle of drowning prevention and chain of drowning survival. Int. J. Aquat. Res. Educ..

[15] Bugeja L., Franklin R.C. (2013). An analysis of stratagems to reduce drowning deaths of young children in private swimming pools and spas in Victoria, Australia. Int. J. Inj. Control Saf. Promot..

[16] Saunders C., Sewduth D., Naidoo N. (2018). Keeping our heads above water: A systematic review of fatal drowning in South Africa. S. Afr. Med. J..

[17] Sarrassat S., Mrema S., Tani K., Mecrow T., Ryan D., Cousens S. (2018). Estimating drowning mortality in Tanzania: A systematic review and meta-analysis of existing data sources. Inj. Prev..

[18] Moher D., Liberati A., Tetzlaff J., Altman D.G. (2009). Preferred reporting items for systematic reviews and meta-analyses: The PRISMA statement. Ann. Intern. Med..

[19] Sirriyeh R., Lawton R., Gardner P., Armitage G. (2012). Reviewing studies with diverse designs: The development and evaluation of a new tool. J. Eval. Clin. Pract..

[20] Streatfield P.K., Khan W.A., Bhuiya A., Hanifi S.M., Alam N., Diboulo E., Niamba L., Sié A., Lankoandé B., Millogo R. (2014). Mortality from external causes in Africa and Asia: Evidence from INDEPTH Health and Demographic Surveillance System Sites. Glob. Health Action.

[21] Wu Y., Huang Y., Schwebel D.C., Hu G.Q. (2017). Unintentional Child and Adolescent Drowning Mortality from 2000 to 2013 in 21 Countries: Analysis of the WHO Mortality Database. Int. J. Environ. Res. Public Health.

[22] Ching-Yih L., Yi-Fong W., Tsung-Hsueh L., Ichiro K. (2015). Unintentional drowning mortality, by age and body of water: An analysis of 60 countries. Inj. Prev..

[23] Davis S., Smith L.S. (1982). Alcohol and drowning in Cape Town. A preliminary report. S. Afr. Med. J..

[24] Davis S., Smith L.S. (1985). The epidemiology of drowning in Cape Town--1980-1983. S. Afr. Med. J..

[25] Donson H., Van Niekerk A. (2013). Unintentional drowning in urban South Africa: A retrospective investigation, 2001–2005. Int. J. Inj. Control Saf. Promot..

[26] Morris N.K., Du Toit-Prinsloo L., Saayman G. (2016). Drowning in Pretoria, South Africa: A 10-year review. J. Forensic Leg. Med..

[27] Meel B.L., Meel B.L. (2008). Drowning deaths in Mthatha area of South Africa. Med. Sci. Law.

[28] Knobel G.J., De Villiers J.C., Parry C.D.H., Botha J.L. (1984). The causes of non-natural deaths in children over a 15-year period in greater Cape Town. S. Afr. Med. J..

[29] Mendes J.F., Mathee A., Naicker N., Becker P., Naidoo S. (2011). The prevalence of intentional and unintentional injuries in selected Johannesburg housing settlements. S. Afr. Med. J..

[30] Joanknecht L., Argent A.C., van Dijk M., van As A.B. (2015). Childhood drowning in South Africa: Local data should inform prevention strategies. Pediatr. Surg. Int..

[31] Kibel S.M., Joubert G., Bradshaw D. (1990). Injury-related mortality in South African children, 1981–1985. S. Afr. Med. J..

[32] Lerer L.B., Matzopoulos R.G., Phillips R. (1997). Violence and injury mortality in the Cape Town metropole. S. Afr. Med. J..

[33] Pretorius K., Van Niekerk A. (2015). Childhood psychosocial development and fatal injuries in Gauteng, South Africa. Child Care Health Dev..

[34] Meel B.L. (2017). Incidence of unnatural deaths in Transkei sub-region of South Africa (1996–2015). S. Afr. Fam. Pract..

[35] Burrows S., van Niekerk A., Laflamme L. (2010). Fatal injuries among urban children in South Africa: Risk distribution and potential for reduction. Bull. World Health Organ..

[36] Matzopoulos R., Prinsloo M., Pillay-van Wyk V., Gwebushe N., Mathews S., Martin L.J., Laubscher R., Abrahams N., Msemburi W., Lombard C. (2015). Injury-related mortality in South Africa: A retrospective descriptive study of postmortem investigations. Bull. WHO.

[37] Mathews S., Martin L.J., Coetzee D., Scott C., Naidoo T., Brijmohun Y., Quarrie K. (2016). The South African child death review pilot: A multiagency approach to strengthen healthcare and protection for children. S. Afr. Med. J..

[38] Groenewald P., Bradshaw D., Neethling I., Martin L.J., Dempers J., Morden E., Zinyakatira N., Coetzee D. (2016). Linking mortuary data improves vital statistics on cause of death of children under five years in the Western Cape Province of South Africa. Trop. Med. Int. Health.

[39] Reid A.E., Hendricks M.K., Groenewald P., Bradshaw D. (2016). Where do children die and what are the causes? Under-5 deaths in the Metro West geographical service area of the Western Cape, South Africa, 2011. S. Afr. Med. J..

[40] Garrib A., Herbst A.J., Hosegood V., Newell M.L. (2011). Injury mortality in rural South Africa 2000 - 2007: Rates and associated factors. Trop. Med. Int. Health.

[41] Erasmus E., Robertson C., van Hoving D.J. (2018). The epidemiology of operations performed by the National Sea Rescue Institute of South Africa over a 5-year period. Int. Marit. Health.

[42] Flisher A.J., Joubert G., Yach D. (1992). Mortality from external causes in South African adolescents, 1984–1986. S. Afr. Med. J..

[43] Saunders C.J., Adriaanse R., Simons A., van Niekerk A. (2018). Fatal drowning in the Western Cape, South Africa: A 7-year retrospective, epidemiological study. Inj. Prev..

[44] Gelaye K.A., Tessema F., Tariku B., Abera S.F., Gebru A.A., Assefa N., Zelalem D., Dedefo M., Kondal M., Kote M. (2018). Injury-related gaining momentum as external causes of deaths in Ethiopian health and demographic surveillance sites: Evidence from verbal autopsy study. Glob. Health Action.

[45] Weldearegawi B., Ashebir Y., Gebeye E., Gebregziabiher T., Yohannes M., Mussa S., Berhe H., Abebe Z. (2013). Emerging chronic non-communicable diseases in rural communities of Northern Ethiopia: Evidence using population-based verbal autopsy method in Kilite Awlaelo surveillance site. Health Policy Plan..

[46] Ohene S.A., Tettey Y., Kumoji R. (2010). Injury-related mortality among adolescents: Findings from a teaching hospital’s post mortem data. BMC Res. Notes.

[47] Ossei P.P.S., Ayibor W.G., Agagli B.M., Aninkora O.K., Fuseini G., Oduro-Manu G., Ka-Chungu S. (2019). Profile of unnatural mortalities in Northern part of Ghana; a forensic-based autopsy study. J. Forensic Leg. Med..

[48] Chasimpha S., McLean E., Chihana M., Kachiwanda L., Koole O., Tafatatha T., Mvula H., Nyirenda M., Crampin A.C., Glynn J.R. (2015). Patterns and risk factors for deaths from external causes in rural Malawi over 10 years: A prospective population-based study Health behavior, health promotion and society. BMC Public Health.

[49] Purcell L., Mabedi C.E., Gallaher J., Mjuweni S., McLean S., Cairns B., Charles A. (2017). Variations in injury characteristics among paediatric patients following trauma: A retrospective descriptive analysis comparing pre-hospital and in-hospital deaths at Kamuzu Central Hospital, Lilongwe, Malawi. Malawi Med. J..

[50] Seleye-Fubara D., Nicholas E.E., Esse I. (2012). Drowning in the Niger Delta region of Nigeria: An autopsy study of 85 cases. Niger. Postgrad. Med. J..

[51] Osime O.C., Ighedosa S.U., Oludiran O.O., Iribhogbe P.E., Ehikhamenor E., Elusoji S.O. (2007). Patterns of trauma deaths in an accident and emergency unit. Prehospital Disaster Med..

[52] Olatunya O.S., Isinkaye A.O., Oluwadiya K.S. (2015). Profile of non-accidental childhood injury at a tertiary hospital in south-west Nigeria. J. Trop. Pediatr..

[53] Lett R.R., Kobusingye O.C., Ekwaru P. (2006). Burden of injury during the complex political emergency in northern Uganda. Can. J. Surg..

[54] Kobusingye O., Tumwesigye N.M., Magoola J., Atuyambe L., Olange O. (2017). Drowning among the lakeside fishing communities in Uganda: Results of a community survey. Int. J. Inj. Control Saf. Promot..

[55] Kobusingye O., Guwatudde D., Lett R. (2001). Injury patterns in rural and urban Uganda. Inj. Prev..

[56] Koné S., Fürst T., Jaeger F.N., Esso E.L.J.C., Baïkoro N., Kouadio K.A., Adiossan L.G., Zouzou F., Boti L.I., Tanner M. (2015). Causes of death in the Taabo health and demographic surveillance system, Cǒte d'Ivoire, from 2009 to 2011. Glob. Health Action.

[57] Odhiambo F.O., Beynon C.M., Ogwang S., Hamel M.J., Howland O., van Eijk A.M., Norton R., Amek N., Slutsker L., Laserson K.F. (2013). Trauma-related mortality among adults in rural western Kenya: Characterizing deaths using data from a health and demographic surveillance system. PLoS ONE.

[58] Mamady K., Yao H., Zhang X., Xiang H., Tan H., Hu G. (2012). The injury mortality burden in Guinea. BMC Public Health.

[59] Moshiro C., Mswia R., Alberti K.G.M.M., Whiting D.R., Unwin N., Setel P.W. (2001). The importance of injury as a cause of death in sub-Saharan Africa: Results of a community-based study in Tanzania. Public Health.

[60] Grainger C.R. (1985). Drowning accidents in the Seychelles. J. R. Soc. Health.

[61] Chitiyo M.E. (1974). Causes of unnatural adult deaths in the Bulawayo area. Cent. Afr. J. Med..

[62] World Health Organization ICD-11 for Mortality and Morbidity Statistics. https://icd.who.int/browse11/l-m/en#/http%3a%2f%2fid.who.int%2ficd%2fentity%2f128104623.

[63] Wikimedia Commons Blank Map of Africa. File: Blank Map-Africa.svg.

[64] Manuele F.A. (2005). Risk assessment and hierarchies of control. Prof. Saf..

[65] Cinnamon J., Schuurman N. (2010). Injury surveillance in low-resource settings using Geospatial and Social Web technologies. Int. J. Health Geogr..

[66] Lukaszyk C., Ivers R.Q., Jagnoor J. (2018). Systematic review of drowning in India: Assessment of burden and risk. Inj. Prev..

[67] World Health Organization Global Health Estimates (GHE) 2016. https://www.who.int/healthinfo/global_burden_disease/estimates/en/.

[68] Driscoll T.R., Harrison J.A., Steenkamp M. (2004). Review of the role of alcohol in drowning associated with recreational aquatic activity. Inj. Prev..

[69] Institute for Health Metrics and Evaluation (IHME) (2017). GBD Compare Data Visualization. https://vizhub.healthdata.org/gbd-compare/.

[70] Peden A.E., Franklin R.C., Clemens T. (2019). Exploring the burden of fatal drowning and data characteristics in three high income countries: Australia, Canada and New Zealand. BMC Public Health.

[71] Cenderadewi M., Franklin R.C., Peden A.E., Devine S. (2019). Pattern of intentional drowning mortality: A total population retrospective cohort study in Australia, 2006–2014. BMC Public Health.

