# Supplementary material for: Epidemiology, Risk Factors and Measures for Preventing Drowning in Africa: A Systematic Review
Source: Medicina (Kaunas). 2019 Sep 25;55(10):637. doi: 10.3390/medicina55100637 (PMC6843779; doi:10.3390/medicina55100637)
Supplement: Supplementary file 1 [file medicina-55-00637-s001.pdf]

medicina

Review

Epidemiology, Risk Factors and Measures for Preventing Drowning in Africa: A Systematic Review

Lauren Miller, Faith O. Alele, Theophilus I. Emeto and Richard C. Franklin

Table S1. Medline search strategy.

**Search Terms**
Drowning
Drownings
Drown*
"immersion injur*"
"submersion injur*"
"bathtub death"
Africa
Algeria OR Angola OR Benin OR Botswana OR "Burkina Faso" OR Burundi OR "Cabo Verde" OR "Cape Verde" OR Cameroon OR Central African Republic OR Chad OR Comoros OR "Democratic Republic of the Congo" OR "Republic of the Congo" OR "Cote d'Ivoire" OR Djibouti OR Egypt OR "Equatorial Guinea" OR Eritrea OR Eswatini OR Swaziland OR Gabon OR Gambia OR Ghana OR Guinea OR "Guinea-Bissau" OR Kenya OR Lesotho OR Liberia OR Libya OR Madagascar OR Malawi OR Mali OR Mauritania OR Mauritius OR Morocco OR Mozambique OR Namibia OR Niger OR Nigeria OR Rwanda OR "Sao Tome and Principe" OR Senegal OR Seychelles OR "Sierra Leone" OR Somalia OR "South Africa" OR "South Sudan" OR Sudan OR Tanzania OR Togo OR Tunisia OR Uganda OR Zambia OR Zimbabwe
1 OR 2 OR 3 OR 4 OR 5 OR 6
7 OR 8
9 AND 10

Table S2. Quality assessment of included studies using the quality assessment tool for studies with diverse designs (QATSDD).

QATSDD Criteria *	1 *	2 *	3 *	4 *	5 *	6 *	7 *	8 *	9 *	10 *	11 *	12 *	Total Score	% of Total Score
Chitiyo, 1974 [71]	0	2	3	0	0	3	2	3	3	3	0	0	19/36	53
Davis et al, 1982 [33]	3	2	3	0	2	3	2	3	3	2	0	0	19/36	53
Knobel et al, 1984 [38]	3	3	3	0	2	3	2	3	3	3	3	0	28/36	78
Davis et al, 1985 [34]	1	1	3	0	2	3	2	3	3	2	0	0	20/36	56
Grainger et al, 1985 [70]	3	1	3	0	2	3	2	3	3	3	3	0	26/36	72
Kibel et al, 1990 [41]	3	2	3	0	2	3	2	3	3	3	3	3	30/36	83
Flisher et al, 1992 [52]	3	2	3	0	2	3	2	3	3	3	3	0	27/36	75
Lerer et al, 1997 [42]	3	2	3	0	2	3	2	3	3	3	3	3	30/36	83
Kobusingye et al, 2001 [65]	3	3	3	3	3	3	3	3	3	3	3	3	36/36	100
Moshiro et al, 2001 [69]	3	3	3	1	3	3	3	3	3	3	3	0	31/36	86
Lett et al, 2006 [63]	3	3	3	3	3	3	3	3	3	3	3	1	34/36	94
Osime et al, 2007 [61]	3	3	3	0	3	3	3	3	3	3	1	0	28/38	78
Meel BL, 2008 [37]	3	3	3	0	3	3	3	3	3	3	0	0	27/36	75
Burrows et al, 2010 [45]	3	3	3	2	3	3	3	3	3	3	3	3	35/36	97
Ohene et al, 2010 [56]	3	3	3	0	3	3	3	3	3	3	3	3	33/36	92
Garrib et al, 2011 [50]	3	3	3	1	3	3	3	3	3	3	3	0	31/36	86
Mendes et al, 2011 [39]	3	2	3	2	3	3	3	3	3	3	3	3	34/36	94
Mamady et al, 2012 [68]	3	3	3	1	3	3	3	3	3	3	3	3	34/36	94
Seleye-Fubara et al, 2012 [60]	3	3	3	0	2	3	3	3	3	3	3	0	29/36	75
Donson et al, 2013 [35]	3	3	3	2	3	3	3	3	3	3	3	3	35/36	97
Odhiambo et al, 2013 [67]	3	3	3	2	3	3	3	3	3	3	3	3	35/36	97
Weldearegawi et al, 2013 [55]	3	3	3	0	3	3	3	3	3	3	3	3	33/36	92
Streatfield et al, 2014 [30]	3	3	3	0	3	3	3	3	3	3	3	0	30/36	83
Chasimpha et al, 2015 [58]	3	3	3	1	3	3	3	3	3	3	3	1	32/36	89
Joanknecht et al, 2015 [40]	3	3	3	0	3	3	3	3	3	3	3	3	33/36	92
Kone et al, 2015 [66]	3	3	3	2	3	3	3	3	3	3	3	0	32/36	89
Lin et al, 2015 [32]	3	3	3	2	3	3	3	3	3	3	3	3	35/36	97
Matzopoulos et al, 2015 [46]	3	3	3	2	3	3	3	3	3	3	3	0	32/36	89
Olatunya et al, 2015 [62]	3	3	3	0	3	3	3	3	3	3	3	3	33/36	92
Pretorius et al, 2015 [43]	3	3	3	2	3	3	3	3	3	3	3	0	32/36	89
Groenewald et al, 2016 [48]	3	3	3	0	3	3	3	3	3	3	3	0	30/36	83
Mathews et al, 2016 [47]	3	3	3	1	3	3	3	3	3	3	3	3	34/36	94
Morris et al, 2016 [36]	3	3	3	1	3	3	3	3	3	3	3	0	31/36	86
Reid et al, 2016 [49]	3	3	3	0	3	3	3	3	3	3	3	1	31/36	86
Kobusingye et al, 2017 [64]	3	3	3	3	3	3	3	3	3	3	3	3	36/36	100
Meel BL, 2017 [44]	3	3	3	1	3	3	3	3	3	3	3	3	35/36	97
Purcell et al, 2017 [59]	3	3	3	0	3	3	3	3	3	3	3	3	33/36	92

Wu et al, 2017 [31]	3	3	3	2	3	3	3	3	3	3	3	3	35/36	97
Erasmus et al, 2018 [51]	3	3	3	0	3	3	3	3	3	3	3	3	33/36	92
Gelaye et al, 2018 [54]	3	3	3	0	3	3	3	3	3	3	3	0	30/36	83
Saunders et al, 2018 [53]	3	3	3	3	3	3	3	3	3	3	3	3	36/36	100
Ossei et al, 2019 [57]	3	3	3	3	3	3	3	3	3	3	3	0	33/36	92

* **QATSDD Criteria:** (1) Theoretical framework; (2) Aims/objectives; (3) Description of research setting; (4) Sample size; (5) Representative sample of target group; (6) Procedure for data collection; (7) Rationale for choice of data collection tool(s); (8) Detailed recruitment data; (9) Fit between research question and method of data collection (Quantitative only); (10) Fit between research question and method of analysis (Quantitative only); (11) Good justification for analytical method selected; (12) Strengths and limitations. QATSDD rating scale: 0 = not at all; 1 = very slightly; 2 = moderately; 3 = complete.

PRISMA 2009 Flow Diagram

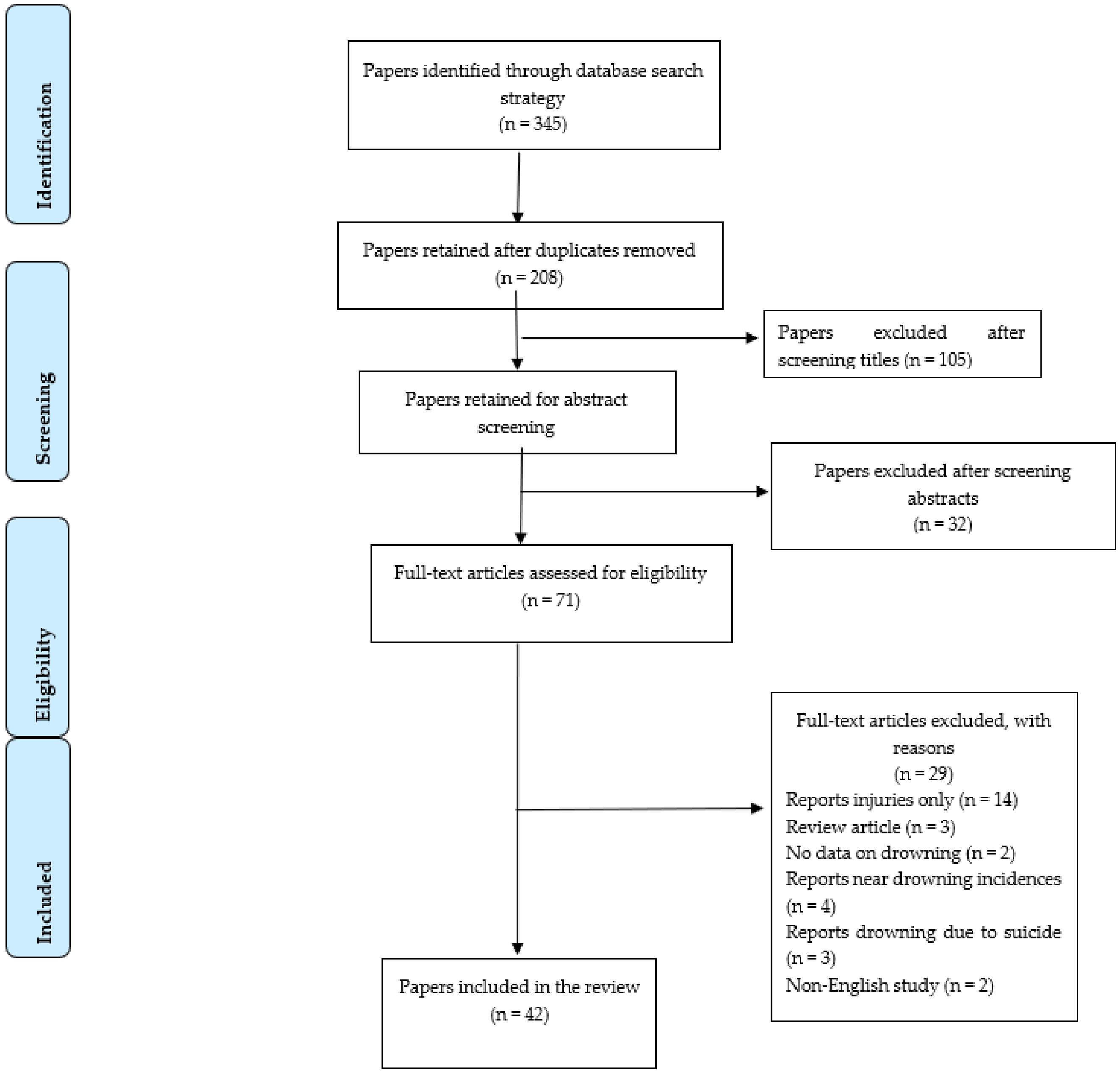

Figure S1. Schematic of study inclusion.
